# Physicochemical degradation of phycocyanin and means to improve its stability: A short review

**DOI:** 10.1016/j.jpha.2021.12.005

**Published:** 2021-12-28

**Authors:** Aïda Adjali, Igor Clarot, Zilin Chen, Eric Marchioni, Ariane Boudier

**Affiliations:** a Université de Lorraine, CITHEFOR, F-54000, Nancy, France; b Key Laboratory of Combinatorial Biosynthesis and Drug Discovery, Ministry of Education, Hubei Province Engineering and Technology Research Center for Fluorinated Pharmaceuticals, and Wuhan University School of Pharmaceutical Sciences, Wuhan, 430071, China; c State Key Laboratory of Transducer Technology, Chinese Academy of Sciences, Beijing, 100080, China; d Université de Strasbourg, CNRS, IPHC UMR 7178, F-67000, Strasbourg, France

**Keywords:** *Arthrospira platensis*, Spirulina, Phycocyanin stability, Preservatives, Encapsulation

## Abstract

The cyanobacterium *Arthrospira platensis*, spirulina, is a source of pigments such as phycobiliprotein and phycocyanin. Phycocyanin is used in the food, cosmetics, and pharmaceutical industries because of its antioxidant, anti-inflammatory, and anticancer properties. The different steps involved in extraction and purification of this protein can alter the final properties. In this review, the stability of phycocyanin (pH, temperature, and light) is discussed, considering the physicochemical parameters of kinetic modeling. The optimal working pH range for phycocyanin is between 5.5 and 6.0 and it remains stable up to 45 °C; however, exposure to relatively high temperatures or acidic pH decreases its half-life and increases the degradation kinetic constant. Phycobiliproteins are sensitive to light; preservatives such as mono- and di-saccharides, citric acid, or sodium chloride appear to be effective stabilizing agents. Encapsulation within nano- or micro-structured materials such as nanofibers, microparticles, or nanoparticles, can also preserve or enhance its stability.

## Highlights

• Phycocyanin is in great demand for industrial application.
• Phycocyanin is sensitive to pH, temperature, and light.
• Optimal stability occurs between pH 5.5–6.0 and at temperatures <45 °C in the dark.
• The use of preservatives or its encapsulation with polymers enhances its stability.

## Graphical abstract

**Figure:**
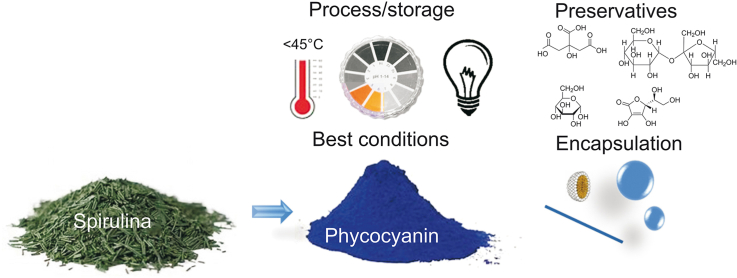

## 1 Introduction

**Fig. 1:**
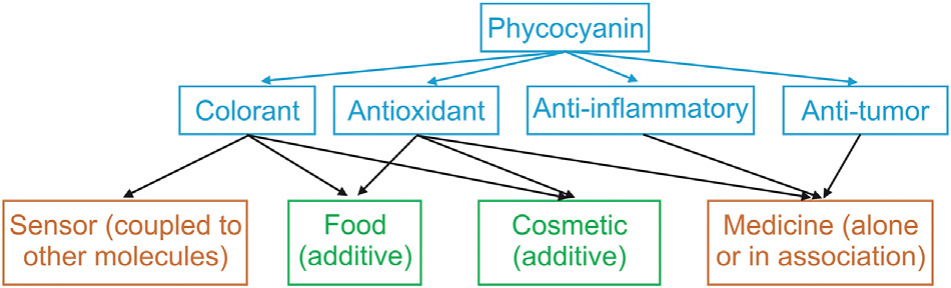
Applications of phycocyanin: green represents already marketed products whereas orange represents products under development.

Spirulina or *Arthrospira platensis* (*A.* *platensis*) is a microscopic cyanobacterium that is considered to be an environmental friendly microalga. This organism is characterized by its rapid growth and consumption of little energy and water per kilogram [1]. This cyanobacterium has received significant attention because of its high protein content, which accounts for up to 70% of its dry mass. Among the various nutrients (vitamins A, B12, B6, C, D, beta-carotene, and minerals) that constitute spirulina, phycocyanin, a phycobiliprotein responsible for the blue-green color of spirulina, represents up to 20% of the dry mass of total cellular proteins [2,3]. The different applications of phycocyanin are shown in Fig. 1.

In terms of nutrition, this cyanobacterium globally represents a source of interest because of its high protein and antioxidant contents, which possess reactive oxygen scavenging and metal chelating activities [4]. Metal chelating activity eliminates toxins such as heavy metals (iron, copper, and cadmium) by ion exchange and chelation in 5-membered chelated rings [5]. Antioxidant and detoxification properties are also exploited for cosmetic applications in masks, creams, and gels.

Phycobiliproteins, including phycocyanin, are used as a natural blue dye [6] in food (chewing gum, dairy products, ice, and jellies), cosmetics (lipsticks), and in medicine as biochemical tracers in immunoassays because of their fluorescent properties [7].

Phycocyanin has the potential for drug development. It exerts anti-tumor effects and exhibits apoptotic characteristics such as DNA fragmentation, nuclear condensation, membrane bubble formation, and cell shrinkage [8]. In addition, its use in combination with anti-inflammatory (piroxicam [9]) and anticancer (topotecan [10] and doxorubicin [11]) drugs appears to be promising, even in multi-drug resistant cells [11]. The antioxidant activity of phycocyanin is also relevant in the alleviation of many diseases, including inflammation, cancer, and other disorders caused by oxidative stress [12]. The bioactivity of phycocyanin and other compounds extracted from algae has been extensively reviewed in literature [8,13,14], which is beyond the scope of this review.

Extraction of phycocyanin from *A. platensis* as a protein-pigment complex appears to be easy because of its high solubility in water. Various production, separation, and purification methods have been carried out and extensively covered by other reviews [7,[14], [15], [16], [17], [18], [19]] and are therefore beyond the scope of this paper. However, these methods are considered to present a major obstacle with respect to the stability of phycocyanin during the extraction and purification processes. This molecule appears to be highly sensitive to environmental stress, particularly temperature, pH, and light [6,20]. This instability also limits its use in food and cosmetic domains for the development of products because it causes precipitation and partial discoloration by changing from blue to green or by total discoloration. This may also result in unwanted effects, such as loss of antioxidant capacity [21]. Therefore, numerous stability studies have been performed to determine the optimal conditions for use by evaluating kinetic parameters such as the order of the reaction, the kinetic constant, or the half-life as a function of degradation conditions. These studies were performed to gather information about the change in food quality, as previously described for other antioxidants [22]. To avoid phycocyanin degradation and to improve its shelf-life, the use of stabilizing agents or direct encapsulation in different types of particles was explored. These methods are widely used in food products and cosmetic formulations. This review discusses the experimental methods and physicochemical parameters used to study the in vitro degradation of phycocyanin. This will provide practical keys to experimentations to define the conditions for its proper use or to test new stabilizers. The different stabilization techniques (via preservatives or formulation processes) will also be presented, which is original from other review articles [23].

## 2 Structural, physical, and chemical characterizations of phycocyanin

**Fig. 2:**
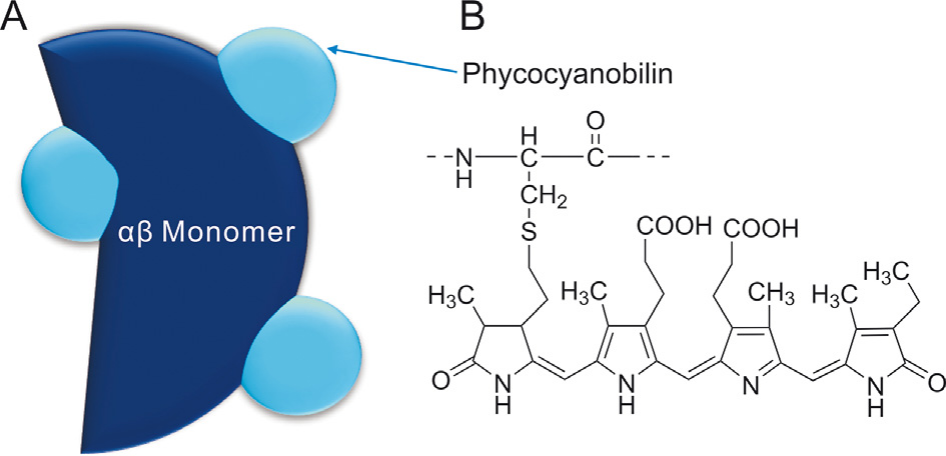
Structure of (A) αβ monomer of phycocyanin and (B) phycocyanobilin.

Phycocyanin (220 kDa) is composed of a polypeptide chain and chromophores called phycocyanobilin (Fig. 2). The polypeptide chain comprises α- and β-subunits with molecular masses between 18 and 20 kDa [24]. The two subunits form monomers (αβ), which organize into trimer (αβ)_3_ and further into hexamers (αβ)_6_. Each monomer contains three chromophores, which are positioned at the Cys-84 residues of subunit α, and Cys-84 and Cys-155 of the β subunit.

UV-vis absorbance is conventionally used to evaluate the purity and degradation of phycocyanin because the protein is characterized by a maximum absorbance at 620 nm with two weaker bands at 360 nm and 650 nm [25]. The ratio of absorbance at 620 nm/absorbance at 280 nm (*A*_620_/*A*_280_) is commonly used to evaluate phycocyanin purity after extraction and purification. This highlights the purity of the protein compared to that of the contaminating proteins (non-specific absorbance at 280 nm). In general, a purity ratio between 0.7 and 3.9 indicates that phycocyanin is provided as food grade and a higher purity ratio indicates reagent/analytical grade [16]. High purity can be obtained when the ratio is >4 [26]. The *A*_620_/*A*_280_ ratio is also used to monitor protein denaturation [25,27]. This is explained by a change in conformation of the tetrapyrrole chromophores due to the de-folding of the protein. This imposes a particular conformation of the chromophore inducing a modification in the absorbance ratio [27].

## 3 Degradation studies

### 3.1 Experimental conditions

Table 1 [[6], [28], [29], [30], [31], [32], [33], [34], [35], [36], [37], [38], [39], [40]] presents an overview of the literature for the experimental conditions used in the degradation studies of phycocyanin.

**Table 1:** Experimental conditions used to study phycocyanin degradation.

Phycocyanin from *Spirulina* except when mentioned			Degradation conditions			Method to study the stability	Model to interpret the results	Focus of the study	Refs.
Supplier or extraction method	Purity (ratio *A*_620_/*A*_280_ when indicated)	Initial concentration and medium	pH	Temperature (°C)	Others				
Extraction in phosphate buffer, centrifugation and filtrations	1.43	1 mg/mL Phosphate citrate buffer (pH 5, 6, 7)	5.0, 5.5, 6.0, 6.5, 7.0	26, 31, 35, 39, 43, 47, 51, 55, 59, 64, 69, 74	–	Spectrophotometry UV-vis	First-order rate law	Stability of phycocyanin and the use of preservatives	[6]
Filtration	0.46	3.68 mg/mL in water	5, 6, 7	50, 53, 55, 57, 60, 62, 65	–	Spectrophotometry UV-vis	First-order rate law	Kinetic study of thermal degradation of phycocyanin	[28]
Supplier GNT International BV	Not indicated	1 mg/mL Phosphate buffer 0.1 M, pH 7.3	–	70, 75, 80	–	Spectrophotometry UV-vis	Sixth-order rate law	Thermal color degradation kinetics of a phycocyanin for short time	[29]
Precipitation with ammonium sulfate and centrifugations	>4.0	0.33 mg/mL Phosphate buffer (0.005 M, pH 4.0 to 8.0) Acetate buffer (pH 4.0 to 5.0)	4.0, 8.0	45, 55, 65	–	Spectrophotometry UV-vis	First-order rate law	Kinetic study of phycocyanin degradation as a function of the pH and the temperature	[30]
Supplier Sigma-Aldrich and GNT International BV	Reagent grade (1.65) and food grades (1.97 and 0.64)	Phosphate buffer 0.1 M, pH 7.0	7.0	50, 65, 80, 90, 100	–	Spectrophotometry UV-vis and circular dichroism	Weibull model	Saccharides and water play an important role in phycocyanin stability	[31]
Precipitation with ammonium sulfate and centrifugations	Reagent grade 1.24	0.5, 2.75, and 5 mg/mL prepared in 50 mM sodium phosphate buffer (pH 6.0)	6.0	60	–	Spectrophotometry UV-vis	None	Stability and antioxidant and antibacterial activity of phycocyanin	[32]
Supplier Linablue-A, Dainippon Ink & Chemicals	–	400 mg/mL in water	2.0, 6.5, 8.0	25, 35, 45	–	Spectrophotometry UV-vis	First-order rate law	Thermal degradation of phycocyanin at various pH	[33]
Sonication and centrifugation from *Nostoc* sp. strain HKAR-2	3.18	0.1 mg/mL in potassium phosphate buffer at pH 7.0	7.0	4, 25, 40	–	Spectrophotometry UV-vis	First-order rate law	Thermal stability in presence of preservatives	[34]
Precipitation with ammonium sulfate and centrifugations	2.25	0.4 mg/mL Phosphate buffer 0.1 M (pH 7.0)	–	0, 35	Presence of urea: 1–10 M	Spectrophotometry UV-vis, differential scanning calorimetry	None	Screening of preservatives to improve the stability of phycocyanin	[35]
Precipitation with ammonium sulfate and centrifugations	1.5	0.4 mg/mL Phosphate citrate buffer pH 3.0, 4.0, 5.0, 6.0, 7.0, 8.0	3.0, 4.0, 5.0, 5.5, 6.0, 7.0, 8.0	25, 30, 35, 40, 45, 50, 55, 60, 65, 70, 75	Light intensity: 50 and 100 μmol/m^2^·s	Spectrophotometry UV-vis	First-order rate law	Stability and antioxidant activity of phycocyanin	[36]
Extracted from Pseudanabaena sp. ABRG5-3, Limnothrix sp. SK1-2-1, Spirulina platensisNIES-39 via centrifugation and freeze-drying and water addition	3.10, 2.14, or 1.76 depending on the strains	4 mg/mL in deionised water then at 0.8 mg/mL in phosphate citrate buffer (0.15 M at pH 4.0, 5.0, 7.0)	4.0, 5.0 7.0	30 or 55	Light intensity: 100 μmol/m^2^·s	Spectrophotometry UV-vis	None	Stability and antioxidant capacity of phycocyanin extracted from various cyanobacteria	[37]
NIES-39 via centrifugation and freeze-drying and water addition									
Ultra-sound and centrifugation	–	Water	–	4, 25, 40	Light: 20 W white fluorescent lamp	HPLC-vis	First-order rate law and second-order with light	Kinetic model to reflect the stability of phycocyanin from ultrasonic extraction process	[38]
Supplier Biotecnología Mexicana de Microalgas S.A. de C.V	0.75	333 μg/mL in distilled water	–	5, 18, 44, 57	Light intensity: 0, 65, 130 μmol/m^2^·s	Spectrophotometry UV-vis	Weibull model and others	Thermo-photostability of phycocyanin	[39]
Centrifugation	–	–	–	25 to 70	Light: UV light 254 nm, 40 W	Spectrophotometry UV-vis and spectrofluorimetry	–	Photostabilization of phycocyanin in presence of biopterin-α-glucoside	[40]

−: no data.

These features have many similarities. Food-grade phycocyanin is generally used for studying its degradation. The degradation is commonly evaluated by UV-vis spectrophotometry based on the spectroscopic properties of the protein. The study variables were the temperature (25–100 °C), pH of the medium (3.0–8.0), and in a few studies, the presence of light. The initial concentration of phycocyanin did not vary in the studies and remained between 0.3 and 3.7 mg/mL. The results were modeled using different mathematical models, and the degradation of phycocyanin generally follows first-order kinetics characterized by a linear decrease in a semi-logarithmic plot of decreasing variables during the reaction time. Authors commonly determine the kinetic constant (*k*), half-life (*t*_1/2_), and activation energy (*E*_a_). However, some studies monitored the results using a reaction order of *n* = 6 or a Weibull model [29,31,39]. Böcker et al. [29] showed low correlation coefficients with first-order kinetics. Consequently, their degradation profile was modeled using a progressive approach with a non-linear decrease and a reaction order of *n* = 6. This deviation may be related to different treatment parameters and differences in the source of phycocyanin, as suggested by the authors. This study examined a food coloring formula containing 15.7% (*m*/*m*) phycocyanin. According to the authors, this formulation is relevant for applications in the food industry compared to conditions reported in other publications. The high reaction order (*n* = 6) was explained as an empirical reaction order that describes the decrease in coloring activity better than other tested reaction orders. A high reaction order indicates that other parallel reactions, consecutive reactions, or intermediate products are formed, influencing the course of the reaction [29]. In another study, the Weibull equation showed a better fit in the description of the decrease in the absorbance of food-grade phycocyanin and reagent [31] compared to the first-order kinetic model used in other studies [6,28,30]. The authors suggested that differences in the origin of the samples and the methodologies of the heat treatment may be the reason for the best fit made by the Weibull model with respect to the first-order model. Table 2 [6,28,30,36] presents some detailed results after fitting the data to the first-order kinetic model obtained in the studies.

**Table 2:** Some extracted results (selected according to compare almost same temperatures) after fitting the data to the first-order kinetic model.

T (°C)	pH 5.0			pH 6.0			pH 7.0			Refs.
	*k* (min^−1^)	*t*_1/2_ (min)	*E*_a_ (kJ/mol)	*k* (min^−1^)	*t*_1/2_ (min)	*E*_a_ (kJ/mol)	*k* (min^−1^)	*t*_1/2_ (min)	*E*_a_ (kJ/mol)	
47	0.006 ± 0.003	116.5 ± 22.3	100.3^a^	0.0022 ± 0.0001	309.4 ± 12.0	120.32^a^	0.0054 ± 0.0005	128.6 ± 13.3	116.68^a^	[6]
55	0.0202 ± 0.0013	34.4 ± 2.2		0.0075 ± 0.0018	92.7 ± 23.2		0.0146 ± 0.0034	47.5 ± 8.9		
69	0.0928 ± 0.0102	7.5 ± 1.7		0.0481 ± 0.0064	14.5 ± 4.2		0.1156 ± 0.0328	6.0 ± 1.7		
74	0.1068 ± 0.0232	6.5 ± 1.4		0.0707 ± 0.0030	9.7 ± 1.6		0.1361 ± 0.0256	5.3 ± 1.0		
50	5.99 × 10^–4^	1.155	387.14^a^	4.79 × 10^–4^	1.444	559.96^a^	1.19 × 10^–3^	0.577	202.70^a^	[28]
55	1.20 × 10^–2^	0.057		2.99 × 10^–3^	0.231		5.98 × 10^–3^	0.115		
57	2.40 × 10^–2^	0.028		3.59 × 10^–2^	0.019		8.38 × 10^–3^	8.250		
60	6.59 × 10^–2^	10.500		0.220	3.033		0.01	6.416		
45	9.58 × 10^–4^	–	92.048 ^b^	3.23 × 10^–4^	–	154.48 ^b^	1.14 × 10^–3^	–	158.99 ^b^	[30]
55	2.63 × 10^–3^	–		2.51 × 10^–3^	–		4.55 × 10^–3^	–		
65	8.98 × 10^–3^	–		1.14 × 10^–2^	–		4.37 × 10^–2^	–		
50	0.0022 ± 0.0000	321.3 ± 5.5	78.5^a^	0.0014 ± 0.0000	495.0 ± 9.9	103.1^a^	0.0028 ± 0.0001	243.6 ± 10.6	83.3^a^	[36]
55	0.0036 ± 0.0000	193.0 ± 0.6		0.0027 ± 0.0002	253.9 ± 17.1		0.0058 ± 0.0001	119.1 ± 2.4		
65	0.0116 ± 0.0000	58.8 ± 0.1		0.0133 ± 0.0005	52.0 ± 2.0		0.0210 ± 0.0004	33.0 ± 0.7		
75	0.0165 ± 0.0002	42.1 ± 0.4		0.0194 ± 0.0011	35.7 ± 2.0		0.0242 ± 0.0012	28.6 ± 1.4		

Kinetic constant (*k*), half-life (*t*_1/2_) and activation energy (*E*_a_) at pH 5.0, 6.0 and 7.0. ^a^*E*_a_ is calculated using Arrhenius' equation: $k=A\times e^{-\frac{E_{\text{a}}}{RT}}$ with *k* as the kinetic constant, A as the pre-exponential factor, *E*_a_ as the activation energy, *R* as the universal gas constant, and *T* as the absolute temperature and using the slope of the graph $\ln\;K=f\left(\frac{1}{T}\right)$. For each study a linear relation was obtained with a correlation coefficient >0.903. ^b^ Indicated in the article.

### 3.2 Effect of phycocyanin purity

The degradation kinetics of reagent grade and food-grade phycocyanin were compared [31]. The absorbance values of both showed a decrease with time and temperature, similar to other reports [28]. However, experiments using reactive grade phycocyanin showed significantly lower thermal stability than food-grade phycocyanin (*k* = (1.24 ± 2.2) × 10^13^ vs. (1.1 ± 2.7) × 10^14^ h^−1^, respectively). The explanation of such a phenomenon may rely on the processing of the protein: gentle treatment to best preserve the color when used as a food grade, whereas a more extensive purification and stabilization process, including lyophilization, when used as a reactive grade. This may affect the native structure of the protein, which is sensitive to environmental stress [31].

### 3.3 Effect of temperature

Various studies have investigated the effect of temperature on the breakdown of phycocyanin. At values close to room temperature (25–47 °C), phycocyanin solution degrades very slowly (*t*_1/2_ = 309.4 ± 12.0 min at 47 °C and pH = 6) [6]. Some authors have reported the absence of degradation up to 45 °C [36]. Between 47 and 69 °C, the degradation rate was higher in relation to the reported *t*_1/2_ (14.5 ± 4.2 min and at pH = 6) (Table 2) [6,28,30,36]. These profiles have also been reported in various studies [[28], [29], [30],^37^]. At temperatures above 70 °C, the denaturation of the protein was accelerated (*t*_1/2_ = 9.7 ± 1.6 min at 74 °C and pH = 6) [6], consistent with other studies [29,36]. The values of activation energies reported in Table 2 are in the range of several hundred kJ/mol, showing a strong temperature dependence of the degradation of phycocyanin. From these reports, a temperature less than 45 °C is optimal for preserving phycocyanin stability.

### 3.4 Effect of pH

Acidity of a medium is an important element that destabilizes phycocyanin, leading to its degradation. pH of the medium also modifies the spectral properties and color of the protein. Phycocyanin solution at neutral pH is perceived as blue and at acidic pH as green. Exploiting this sensitivity, pH indicator polylactic acid/polyethylene oxide ultrafine fibers containing phycocyanin was developed [41]. This is important for food industries. Phycocyanin solutions under acidic pH (3.0 and 4.0 at room temperature) exhibited lower absorbance at 620 nm and stronger absorbance at 280 nm compared to pH 5, 6, and 7 at temperatures of 55 and 65 °C for the same protein concentration. This is explained by the precipitation of proteins [36] and probably by a change in the conformation of the protein. For pH values greater than 4 and up to 6, the chromophore retains its extended geometry; however, when the pH is lower, it folds into a cyclic conformation, modifying its spectral properties [25,42]. The stability can also be dependent on the cyanobacterium strain from which it is extracted [37]. When Spirulina was used, the best conditions for protein stability were pH 5.5 and 6.0.

Various studies have demonstrated phycocyanin stability as a function of both pH and temperature (Table 2). At temperatures between 50 and 55 °C and at pH 6.0, phycocyanin remained stable [28]. It was also stable between 57 and 65 °C and at pH 5.0; however, between 50 and 65 °C and at pH 7.0, phycocyanin denatured and became unstable. These results are consistent with those from other studies [6,30,36]. Although several researchers have performed experiments between pH 5.0 and 8.0 to simulate the conditions of some tests (e.g., antioxidant assay, using 1,1-diphenyl-2-picrylhydrazyl or 2,2′-azino-bis(3-ethylbenzothiazoline-6-sulphonic acid), or in cell culture), the results confirmed that the optimal range for maintaining the phycocyanin stability is a pH value between 5.5 and 6.0.

### 3.5 Effect of light

Few studies have explored the impact of light on the degradation of phycocyanin in solution at a constant temperature (25 °C) [[36], [37], [38], [39]]. A methodology to study the relationship between light and temperature on phycocyanin degradation using correlation models was described by Escalante et al. [39]. After exposure to lamps emitting an intensity of 50 and 100 μmol/m^2^·s, the phycocyanin concentration decreased in a dose-dependent manner as a function of time. At the same light intensity, phycocyanin solution adjusted to pH 6.0 showed less degradation than those adjusted to pH 5.0 and pH 7.0. The final protein concentration decreased by approximately 20% after continuous exposure to an intensity of 100 μmol/m^2^·s for 36 h, regardless of the pH of the medium. The authors observed a slightly higher level of degradation after exposure to 100 μmol/m^2^·s total intensity than after exposure to an intensity of 50 μmol/m^2^·s. Similar results were reported by other authors with accelerated degradation when the protein was stored at 40 °C vs. 4 °C [38]. Therefore, the best storage conditions are in the dark.

### 3.6 Recommendations on stability studies

As discussed in Section 2 and Table 1, UV-vis absorbance is conventionally used to evaluate the purity and degradation of phycocyanin. For a wider valorization of this protein, particularly in the pharmaceutical context, stability studies under predefined stressful conditions (e.g., ICH Q1B, CPMP/ICH/279/95 [43] for photostability) will be necessary and separation methods must be used. To further describe the stability and degradation products formed (e.g., peptide sequence composition), high-resolution analytical methods are justified. There are very few studies in the literature that describe systems devoted to phycocyanin stability analysis, such as high-performance liquid chromatography (HPLC) coupled with diode array detection (DAD) [44] or mass spectrometry (MS) [45], which can provide essential information on the structure (characterization) and content of the various impurities formed during the degradation phase. Electrophoretic methods (capillary [46] or gel [47]) have also been described for phycocyanin analysis, and many developments are expected in the future for stability testing.

## 4 Methods to improve phycocyanin stability

Preservatives are necessary to ensure that the manufactured food remains safe and intact for a long time (shelf life). Salts, sugars, and vinegar have been used for centuries as food preservatives. As described above, phycocyanin is sensitive to environmental stress. The degradation of the protein fraction significantly impacts the maintenance of color and bioactivity of phycocyanin [48], which are major elements in industries. This explains the use of preservatives and the encapsulation in particulate forms to preserve its color and prevent its denaturation. In this section, only studies describing results on protein stability were reviewed.

### 4.1 Use of preservatives

Preservatives are used to enhance the stability of phycocyanin (Table 3 [6,28,31,32,[34], [35], [36],^40^,[49], [50], [51]]).

**Table 3:** Chemical compounds used to improve the storage.

Phycocyanin			Conditions		Preservatives	Methods to study the stability	Model to interpret the results	Main result of the study	Refs.
Supplier or extraction method	Purity (ratio *A_620_/A_280_* when indicated)	Initial concentration and medium	pH	T (°C)					
Extraction in phosphate buffer, centrifugation and filtrations	1.43	1 mg/mL Phosphate citrate buffer (pH 5, 6, 7)	7.0	60	Glucose 2.5%–40% (*m*/*V*), saccharose 2.5%–40% (*m*/*V*), sorbitol 20% (*m*/*V*), sodium chloride 2.5%–20% (*m*/*V*), ascorbic acid 2.5% (*m*/*V*), citric acid 2.5% (*m*/*V*), and sodium azide 2.5% (*m*/*V*)	Spectrophotometry UV-vis and scanning electron microscopy	First-order rate law	Glucose, sucrose and NaCl preserve the protein stability	[6]
Filtration	0.46	3.68 mg/mL in water	pH 5, 6, 7	62	Sorbitol 10%–50% (*m*/*V*)	Spectrophotometry UV-vis	First-order rate law	Sorbitol improves the stability of phycocyanin even at 10%	[28]
Supplier Sigma-Aldrich and GNT International BV	Reagent grade (1.65) and food grades (1.97 and 0.64)	Phosphate buffer 0.1 M, pH 7.0	–	50, 65, 80, 90, 100	Saccharose 20%, 40%, 70% (*m*/*m*), trehalose 20%, 40% (*m*/*m*)	Spectrophotometry UV-vis and water activity measurement	Weibull model	Sucrose stabilizes more phycocyanin than trehalose	[31]
Precipitation with ammonium sulfate and centrifugations	Reagent grade 1.24	0.5. 2.75 and 5 mg/mL, prepared in 50 mM sodium phosphate buffer (pH 6.0)	6.0	60	Polyethylene glycol-4000 (PEG) or sorbitol and sucrose at a ratio of 1:4 (*m*/*m*) of phycocyanin for stabilizer	Spectrophotometry UV-vis	None	Stability and antioxidant and antibacterial activity of phycocyanin	[32]
Sonication and centrifugation from *Nostoc* sp. strain HKAR-2	3.18	0.1 mg/mL in potassium phosphate buffer at pH 7.0	7.0	4, 25, 40	Calcium chloride, ascorbic acid, sucrose, citric acid, and benzoic acid at 0.5, 2.5, and 5 mM	Spectrophotometry UV-vis	First-order rate law	Benzoic acid is the best preservative	[34]
Precipitation with ammonium sulfate and centrifugations	2.25	0.4 mg/mL Phosphate buffer 0.1 M (pH 7.0)	–	0 and 35	Saccharose (4 g/L), calcium chloride (4 g/L), citric acid (4 g/L) and a combination of previous cited compounds at 4 g/L each or 2 g/L	Spectrophotometry UV-vis and differential scanning calorimetry	None	Citric acid is the best preservative	[35]
Precipitation with ammonium sulfate and centrifugations	1.5	0.4 mg/mL Phosphate citrate buffer pH 5.0, 6.0, 7.0, 8.0	5, 6, 7, 8	65	Saccharose, glucose, and sodium chloride 20% (*m*/*V*)	Spectrophotometry UV-vis	First-order rate law	Sodium chloride stabilizes phycocyanin in a concentration-dependent manner	[36]
Centrifugation	–	–	–	25–70 °C in the presence of UV light 254 nm, 40 W	Biopterin-α-glucoside	Spectrophotometry UV–vis and spectrofluorimetry	–	Photostabilization of phycocyanin in presence of biopterin-α-glucoside	[40]
Extracted	1.0	–	–	55, 60, 65, 70, 75	Sorbitol (50%), saccharose and glucose (20%), sodium chloride (2.5%), and polyethyleneoxide (6%)	Spectrophotometry UV-vis	First-order rate law	Enhancement of phycocyanin stability when using glucose or sorbitol or with nanofibers	[49]
Centrifugation and filtrations	1.4	0.02–1.3 mg/mL in phosphate buffer	5, 7, 9	50, 60, 70, 80	Conventional honey, honey from *Leptospermum scoparium*, fructose (62%, *m*/*V*), glucose (37%, *m*/*V*), and saccharose (54%, *m*/*V*)	Spectrophotometry UV-vis	None	Fructose is the best preservative	[50]
Supplied from CV Neoalgae (Sukoharjo, Indonesia)	–	1 mg/mL in citrate buffer (pH 6.0)	6.0	40, 60, 80	Glucose, sucrose, and fructose 10%–15% (*m*/*V*)	Spectrophotometry UV-vis	First-order rate law	Fructose preserves phycocyanin color	[51]

The chemical structure and the concentrations of the preservatives used are important because the resulting mixture must remain safe for humans. They must not denature the protein or alter its optical and antioxidant properties. Therefore, some substances are excluded because of safety reasons (e.g., sodium azide and dithiothreitol [6]) or because it is too important to induce protein precipitation (e.g., NaCl 5% [6]). The selected compounds were mono- or di-saccharides (glucose, fructose, saccharose, trehalose, lactose, maltose, and sorbitol), inorganic salts (sodium chloride and calcium chloride), and organic acids (citric acid, ascorbic acid, and benzoic acid). In some studies, the compounds are associated [35]; in others, natural matrices have been tested (conventional honey and honey from Manuka (*Leptospermum scoparium*) [50]), and polymers were also used [32]. A pigment, biopterin-α-glucoside, was also selected to improve the photostability of phycocyanin [40].

Although all the protocols are not completely comparable (in terms of concentration of phycocyanin, composition, and pH of the medium), some conditions appear to be interesting for improving the stability of phycocyanin, particularly towards thermodegradability. Concerning the mono- and di-saccharides, several studies showed that these compounds can improve protein stability [28,31,35,49,50]. Some compounds are better than the others. For example, glucose 20% (*m*/*m*) or sorbitol 50% (*m*/*m*) induced a two-fold increase in the *t*_1/2_ of the protein compared to the control, and each control was better than that of saccharose 20% (*m*/*m*) (*t*_1/2_ nearly similar to the control) [49]. Other researchers stated the importance of the concentrations of mono- or di-saccharides, rather than the compound itself [6]. Therefore, they selected glucose or saccharose, both at 20% (*m*/*m*), and both allowed in preserving more than 62% of the protein after 15 min at 60 °C vs. only 47% without any preservatives [6]. The same study emphasized the use of NaCl at high concentrations (2.5%–20%), which was also tested by Wu et al. [36]. However, the solution became turbid at concentrations of 5% (*m*/*m*) of NaCl. Citric acid preserved 67% phycocyanin after 45 days vs. less than 3% for the control protein without any preservatives [35]. This is explained by its capacity to decrease the pH of the medium from 7 to 6 (optimal pH for the protein) and by its chelating capacity. The use of citric acid has also been described as a stabilizer for other natural proteins (whey protein) [35]. Concerning the photodegradation of phycocyanin, the presence of the pigment biopterin-α-glucoside prevented its degradation and decoloration [40].

### 4.2 Particulate forms

The development of particulate forms is common in the food, cosmetics, and pharmaceutical industries. Studies dealing with the stability of phycocyanin in particulate forms are detailed in Table 4 [[25], [47], [49], [52], [53], [54], [55], [56], [57], [58], [59], [60], [61], [62], [63], [64], [65]].

**Table 4:** Particulate forms used to improve the storage.

Type of formulation and technology employed	Size of the particles (method used)	Main result concerning the stability of phycocyanin	Refs.
Sodium dodecylsulfate micelles	Not indicated but usually few nanometers	Stabilizing effect of the colour even at acidic pH and at high temperatures	[25]
Complexes with α-lactalbumin, β-lactoglobulin, bovine serum albumin, immunoglobulins, or glycomacropeptides	90–120 nm	Improvement of phycocyanin stability in acidified solutions	[47]
Nanofibers produced by electrospinning	Average diameter of 295 nm	Better thermostability of phycocyanin in nanofibers (compared to native protein, enhancement by a 2-fold factor) but in the same range compared to the use of preservatives	[49]
Encapsulated in a hydrogel to dope silica materials	30–40 nm	Improvement of the photostability of phycocyanin	[52]
Chitosan nanoparticles	Average diameter of 457 nm	Enhanced thermal stability when encapsulated (90 min at 50 °C)	[53]
Double emulsion by an aqueous two-phase system	8.8 and 380.5 μm depending on the experimental conditions	Up to 6 months of stability	[54]
Complexes formed with whey and κ-carrageenan	660–3925 nm depending on the experimental conditions	Improvement of the photostability of phycocyanin	[55]
Fibers produced by electrospinning	Mean diameters of 196–542 nm	Enhancement of the thermal stability when encapsulated (increase of the initial temperature of degradation of the protein measured by thermogravimetry)	[56]
Fibers produced by electrospinning	Average diameter of 295–760 nm	Enhanced thermal resistance of phycocyanin	[57]
Microcapsules produced by extrusion	Average diameter of 1.37–2.54 mm	Improvement of the stability against temperature increase in microcapsules. No improvement against light degradation	[58]
Microencapsulation using chitosan or κ-carrageenan	2–4 μm	Improvement of phycocyanin stability when microencapsulated	[59]
Microencapsulation	1.5–316 μm	Better thermal stability when microencapsulated	[60]
Microparticles based on polyvinylalcohol produced by electrospraying	Average diameter of 395 nm	Thermal stability improved in microparticles up to 216 °C	[61]
Microencapsulation using extrusion	1.2 mm	Better stability at high temperatures	[62]
Microencapsulation by emulsion and spry-drying	Not reported	Material used for microencapsulation affects phycocyanin stability	[63]
Cross-linked starches-*C*-phycocyanin composites	Not reported	Stabilization within the composites	[64]
Microencapsulation using extrusion	Not reported	Better stability of phycocyanin towards heat stress linked to a better antioxidant activity	[65]

In addition to the enhancement of the stability of the protein [58], the purpose is to develop active packaging to preserve food because of its antioxidant properties to prolong food *t*_1/2_ and avoid its deterioration [56] and the development of a stabilized phycocyanin-based blue food [25].

The formulation of phycocyanin is a challenge because it is a protein (specific folding that can be modified or even degraded during the process) that is highly soluble in water (difficulty in remaining compartmentalize or encapsulated with low or no uncontrolled release from the particle), and sensitive to pH and temperature (which may limit the use of some formulation protocols). The results from these studies showed that encapsulation efficiency (encapsulated and/or at the surface of the particle) of the protein is high by electrospraying: up to 75% encapsulation was achieved [61], or approximately 100% by microencapsulation [60]. Some authors have indicated that phycocyanin is sometimes encapsulated within the particle, whereas the protein is also located at the interface between the external medium and the particle [56]. There has been no report on the possible degradation of proteins during the formulation processes.

The tested formulation protocols did not modify the antioxidant properties of the protein [56,60,61], and all the reported studies showed enhanced stability of the encapsulated protein with respect to temperature and pH modifications. However, the improved thermostability of phycocyanin in nanofibers was in the same range as that obtained with the use of preservatives [49].

In addition, in vitro release studies of polymeric microcapsules encapsulating phycocyanin in conditions mimicking gastric and intestinal fluids were performed. The results demonstrated protection of the protein from acidic gastric fluid and a sustained release profile in simulated intestinal fluid as a function of the used polymer (i.e., alginate, chitosan, and carrageenan) [58,59]. This emphasizes the role of the formulation in protecting the protein from the intestinal fluids.

Although these studies are preliminary, they are interesting in terms of the properties of phycocyanin and for future use in the food industry and other domains such as therapeutics, cosmetics, and sensors.

### 4.3 Recommendations on the use of preservatives and development of particulate forms

Research on preservatives is devoted to the study of the intermolecular interactions between the protein and the preservatives [31]. However, apart from this fundamental knowledge, other parameters must be cited. The regulation of additives is complex and differs from one country to another [66]. Many additives are not devoid of side effects. For further information, for example, the reader is referred to the document edited by the European Commission describing the side effects originating from excipients contained in medicines [67]. Similarly, consumers are increasingly questioning additives in food even though they are necessary to ensure the quality of products. Studies that consider this question showed that consumers are generally more sensitive to negative arguments than positive ones [[68], [69], [70]]. This is balanced by consumers' level of knowledge about additives and their education level [[68], [69], [70]]. Knowledge regarding food additives has been shown to weaken the risk perception of food safety issues [71]. Other factors must be considered, such as consumers’ knowledge of regulation, their trust in regulators, and their preference for natural products [72].

The encapsulation of phycocyanin in different forms (nanofibers, microparticles, or nanoparticles) is another way to prevent its degradation and may open new opportunities for the use of this high added-value protein. The development of formulations for the food, cosmetic, and pharmaceutical purposes is definitely a challenge. Phycocyanin is hydrophilic and highly sensitive to environmental stresses. The best compromise between the intrinsic parameters of the protein, the chosen excipients/additives, and the selected process is not simple [73,74]. The use of benign processes (with no heat, no ultrasound, and no organic solvent) should be prioritized. Future directions using microfluidic [75] or supercritical fluid technology [76] that have already proved their efficiency in formulating sensitive molecules may emerge.

## 5 Conclusion

Phycocyanin is of particular interest to humans in food, medicine, and cosmetics. However, its sensitivity to environmental conditions may prevent the development of new products. According to previous studies, the optimal conditions required for its stability include a pH between 5.5 and 6.0, low temperature (less than 45 °C), and dark storage conditions. This review suggests the use of separative systems to study the degradation pathways and generated by-products. The addition of stabilizing agents such as mono- or di-saccharides, sodium chloride, or citric acid prevents degradation. Some consideration regarding the use of preservatives is provided towards regulatory aspects and, on more general terms, the acceptance by the population. Finally, as the use of particulate systems appears to be a future direction, some recommendations on the selected processes are included.

## CRediT author statement

**Aïda Adjali:** Writing - Original draft preparation; **Igor Clarot, Zilin Chen**, and **Eric Marchioni:** Writing - Reviewing and Editing; **Ariane Boudier:** Supervision, Writing - Reviewing and Editing.

## Declaration of competing interest

The authors declare that there are no conflicts of interest.
